# Effect of Different Microwave Times on the Nutritional Properties of Glycosylated Soybean 7S

**DOI:** 10.3390/foods14213694

**Published:** 2025-10-29

**Authors:** Tao Cui, Jixin Zhang, Huiqing Xu

**Affiliations:** College of Tourism and Culinary Institute, Yangzhou University, No. 196, Huayang West Road, Hanjiang District, Yangzhou 225127, China; mx120241337@stu.yzu.edu.cn (T.C.); mx120211221@stu.yzu.edu.cn (J.Z.)

**Keywords:** microwave time, soybean 7S protein, digestibility, degree of hydrolysis, amino acid composition

## Abstract

In this study, we investigated the effects of different microwave times (0–120 s) at 900 W power on the nutritional properties of glycosylated soybean 7S protein. The in vitro simulated gastrointestinal digestion revealed that digestibility peaked at 60 s (40.83% gastric, 84.29% intestinal) and was accompanied by a significant increase in hydrolysis. Automatic amino acid analysis indicated superior nutritional quality after 60 s of treatment, as evidenced by optimal amino acid scores (AAS, CS) and the essential amino acid index (EAAI), and by a more favorable amino acid profile for human requirements. However, the proportion of flavor-active amino acids decreased from 83.05% at the initial stage to 53.77%, and the taste activity value (TAV) of fresh amino acids was lower than that of the untreated group, with limited flavor improvement. In conclusion, 60 s is the optimal time for nutritional optimization, which can improve protein digestibility and nutritional value, and the risk of flavor degradation should be considered.

## 1. Introduction

Glycosylated soybean 7S protein serves as a high-quality plant protein source for nutritional supplements, offering irreplaceable nutritional and functional properties. It has a balanced amino acid composition, provides comprehensive nutrition, and has exceptionally high lysine content, effectively compensating for the lysine deficiencies of cereal proteins [1]. Additionally, its flavor-presenting amino acids contribute to a unique taste and aroma. However, traditional processing methods may adversely affect the nutritional and flavor properties of 7S protein [2]. As a fast and convenient green processing technology, microwave heating can significantly change proteins’ structural and functional properties [3,4]. Compared with other green processing technologies, microwave treatment has inherent advantages. It not only achieves rapid, uniform energy transfer through the volume-heating effect, thereby significantly improving efficiency and reducing energy consumption, but also more effectively retains the heat-sensitive nutritional components and functional properties of proteins. After microwave treatment, it is important to examine the changes in the nutritional and flavor properties of glycosylated soybean 7S protein.

In vitro simulated digestibility reflects the degree of protein digestion and absorption in the human body, while the hydrolysis degree indicates the breakdown of protein into amino acids. By simulating the gastrointestinal environment, we can assess the decomposition efficiency and degree of hydrolysis of glycosylated soybean 7S protein, helping us understand how microwave heating alters its molecular structure and functional properties. Ajayi et al. [5] found that microwave-assisted extraction technology at a low power level of 400 W improved the in vitro digestibility of cassava bean protein concentrates, improved their functional and nutritional properties, and expanded their potential applications in food systems. Amino acid composition is a key indicator of protein quality, and nutritional evaluation can determine the value and utilization efficiency of proteins. Analyzing amino acid scores (AAS, CS) and other indicators after microwave treatment helps assess whether the treatment improves the nutritional value of the protein. A.N. et al. [6] analyzed the amino acid composition of heat-processed African yam beans by an amino acid analyzer. It was found that compared with traditional cooking methods, microwave heating was more uniform and shorter, which reduced the degradation of amino acids (especially heat-sensitive amino acids) caused by prolonged heating at high temperatures and better preserved the amino acid content and protein quality, with less impact on the nutritional value of their proteins. Analyzing their content in microwave-treated glycosylated soybean 7S protein helps reveal the mechanism of microwave heating on flavor and nutrition. By controlling microwave time, nutrient loss can be reduced, improving food taste and aroma. This provides theoretical and technical support for the application of glycosylated soybean 7S protein in the food industry and promotes the use of green processing technologies in plant protein utilization.

This experiment investigates the effect of different microwave times on the nutritional properties of glycosylated soybean 7S protein. The results can optimize microwave processing parameters, develop high-bioavailability functional protein products, guide the creation of sports nutrition and easy-to-digest foods, and promote innovative applications of green processing technology in plant protein utilization to meet consumer demands for enhanced nutrition and flavor.

## 2. Materials and Methods

### 2.1. Materials

Low-temperature defatted soybean flour (Zhaoyi Food Co., Ltd., Yantai, China). Pepsin and trypsin were purchased from Fuzhou Fei jing Biotechnology Co., Ltd. (Fuzhou, China). Potassium chloride, potassium dihydrogen phosphate, sodium bicarbonate, sodium chloride (Shanghai GuoYao Group Chemical Reagents Co., Ltd., Shanghai, China). All chemicals and reagents were of analytical grade.

### 2.2. Preparation of Glycosylated Soybean 7S Protein

#### 2.2.1. Preparation of Soybean 7S Protein

Soybean 7S protein was prepared according to the method described by FAO [7] with slight modifications. Defatted soybean powder was dissolved in 0.03 M Tris-HCl buffer (pH 8.5) at a ratio of 1:15 (*w*/*v*) and stirred at 45 °C for 1 h. Subsequently, the solution was centrifuged at 9000× *g* for 30 min at 4 °C. Sodium bisulfite was added to the supernatant to reach a concentration of 0.98 g/L. The solution was then adjusted to pH 6.4 with 2 M HCl, and the treated solution was stored at 4 °C for 12 h. The solution was then centrifuged for 1 h at a ratio of 1:15 (*w*/*v*).

The solution was then adjusted to pH 6.4 with 2 M HCl and stored at 4 °C for 12 h. The supernatant was then collected by centrifugation at 6500× *g* for 20 min at 4 °C. Sodium chloride was added to the solution to a final concentration of 0.25 mol/L. The solution was adjusted to pH 5.5, stirred at room temperature for 30 min, then centrifuged at 9000× *g* for 30 min at 4 °C, and the supernatant collected. The supernatant was collected, and an equal volume of deionized water was added. The pH of the solution was adjusted to 4.8 again. The mixture was centrifuged at 6500× *g*, 4 °C for 20 min, the supernatant was poured off, and the precipitate was washed with distilled water 3 times. The pH was adjusted to 7.5 with 2 M NaOH, and the precipitate was frozen in a refrigerator at −80 °C for more than 4 h. Then, it was put into a freeze dryer and freeze-dried at −55 °C for 48 h to obtain the 7S solid powder. To ensure the purity of the prepared 7S protein, samples were tested using SDS-PAGE electrophoresis [8] and Bradford protein assay [9], which was followed for all subsequent protein concentration measurements.

#### 2.2.2. Preparation of Glucose Glycosylation Products

The glycosylation reaction was performed according to the method of Garrido-Balam Liu Ming et al. [9] with modifications. Soybean 7S protein (Prepared according to Section 2.2.1) was mixed with glucose in a 1:1 (*w*/*w*) ratio, dissolved in 0.01 mol/L phosphate buffer (pH 7.0 ± 0.2), and adjusted to a 25 mg/mL protein concentration. The reaction was stirred at room temperature for 2 h, then heated to 90 °C in a water bath for 3 h. The reaction was immediately cooled in an ice bath, and insoluble material was removed by centrifugation at 2000× *g* for 20 min. The supernatant was dialyzed with a dialysis bag with a molecular weight cutoff of 7–14 kDa for 24 h. It was put into a refrigerator at –80 °C for more than 4 h and freeze-dried with a freeze-dryer at −55 °C for 48 h to obtain the lyophilized 7S-glucose glycosylated sample powder. Then, referring to the literature of Schaafsma [10], the degree of glycosylation of proteins was determined by the OPA method using the degree of grafting as an indicator. The degree of grafting of glycosylated 7S proteins was stabilized at about 35% with the error controlled within 1% to ensure that the samples were consistent in each group.

#### 2.2.3. Microwave Treatment of Samples

The samples were divided into five groups and laid flat in a glass Petri dish at 1 mm ± 0.1 mm thickness. The samples were then subjected to microwave treatment at 900 W for 0, 30, 60, 90, and 120 s, using a microwave oven (PC23M6W). The selection of these time points was based on the method of Zhang et al. [11] And was designed to cover a range from mild to intensive treatment, allowing for the observation of the potential nonlinear effects of microwave energy on the structure and functionality of the glycosylated soybean 7S protein.

### 2.3. In Vitro Simulated Digestion Assay

The in vitro digestion of glycosylated soybean 7S protein under different microwave treatment times was determined by reference to the method of Minekus et al. [12]. Ten mL of sample solution (20 mg/mL) was mixed with 7.5 mL of configured simulated gastric fluid (SGF), 1.6 mL of pepsin solution (25,000 U/mL, ready to use), and 5 µL of 0.3 M CaCl_2_ was added. The pH was adjusted to 3.0 with 1 M HCl, and deionized water was added up to 20 mL. The mixtures were then incubated at 37 °C protected from light (150 rpm) for 2 h. Subsequently, the digested samples were immediately cooled in an ice water bath to inactivate the enzyme and produce gastric digestion products. For the enteric digestion assay, 20 mL of gastric digestion products were mixed with 11 mL of configured simulated intestinal fluid (SIF), 5.0 mL of trypsin solution (800 U/mL, ready to use) and 40 uL of 0.3 M CaCl_2_ were added, the pH of the samples was adjusted to 7.0 with 1 M NaOH, and the volume was mixed up to 40 mL with deionized water. The samples were incubated in darkness at 37 °C (150 rpm) for 2 h. Subsequently, the digested samples were immediately cooled in an ice-water bath to inactivate the enzyme and obtain the intestinal digestion products. The SGF and SIF electrolyte stock solutions were configured according to Table 1.

### 2.4. Measurement of Protein Hydrolysis

The degree of hydrolysis of glycosylated soybean 7S protein and its gastrointestinal digestion products at different microwave times was determined by the ortho-thioaldehyde (OPA) method [13]. 400 μL of sample lysate (1 mg/mL) was taken and mixed well with 3 mL of OPA reagent, and the mixture was allowed to stand at 35 °C for 2 min. The absorbance value of the sample at 340 nm was determined using a spectrophotometer. Distilled water was used instead of the sample solution as a blank control for the determination. A standard curve was plotted using L-serine as the standard to calculate the degree of protein hydrolysis. The OPA reagent was prepared as follows: 80 mg of OPA reagent was dissolved in 2 mL of methanol, and 5 mL of 20% (*w*/*v*) SDS solution, 50 mL of 0.1 M borax solution, and 200 μL of β-mercaptothion were added to bring the OPA reagent to 100 mL.

$$hydrolysis(\%)=\frac{(\mathrm{Serine}{\mathrm{NH}}_{2}-\beta )/\alpha }{h_{\mathrm{tot}}}\times 100\%$$

$$\mathrm{Serine}{\mathrm{NH}}_{2}=\frac{\mathrm{OD}(\mathrm{sample})-\mathrm{OD}(\mathrm{void})}{\mathrm{OD}(\mathrm{standard})-\mathrm{OD}\left(\mathrm{void}\right)}\times 0.9516\times \frac{N\times V}{m\times p}$$

where *α* and *β* are constants, for soy protein, *β* and *α* are 0.97 and 0.342, respectively; SerineNH_2_ is the content of serine amino group per gram of protein, mmoL/g; *h_tot_* = 7.8, is the total number of peptide bonds of soy protein; *N* is the number of times of dilution; *V* is the volume of the supernatant liquid, L; *m* is the mass of the sample, g; and *P* is the protein content of the sample, %.

### 2.5. Amino Acid Determination

Free and hydrolyzed amino acid content was measured by the LA8080 amino acid autoanalyzer [14]. (Tryptophan was not measured).

#### 2.5.1. Sample Preparation

Hydrolysis of amino acids: Protein samples were dissolved entirely in a 1:1 (*V*:*V*) hydrochloric acid solution, hydrolyzed in an electrically heated blast thermostat (110 ± 1 °C) for 22 h. After cooling, the samples were filtered and transferred to a volumetric flask. Then the volume was fixed to 10 mL. 0.05 mL of the filtrate was blown dry under nitrogen, then mixed with hydrochloric acid (0.02 moL/L) and fixed to 2 mL, then filtered by a 0.22 μm porous membrane, and then subjected to free amino acid determination. After filtering through a 0.22 μm microporous membrane, the free amino acid was determined on the machine.

Free amino acid: After mixing the sample, add a 0.02 moL/L hydrochloric acid solution to dissolve it, and then bring the volume to 4 mL. Purification: activate the C18 pretreatment column with 5 mL of methanol and 5 mL of water sequentially. The dissolved sample was added to the small column and filtered through a 0.45 μm filter membrane after passing through the column. Finally, the filtrate was subjected to online determination.

#### 2.5.2. Chromatographic Conditions for Amino Acid Analysis

Sulfonic acid type cation resin was used for cation exchange. The reaction temperature was 135 ± 5 °C. The flow rate of buffer was 0.40 mL/min, the flow rate of colorant solution was 0.35 mL/min, and the injection volume was 20 μL. The reaction with ninhydrin was detected at two wavelengths (570 nm and 440 nm, respectively). The final amino acid content was expressed as g/100 g protein [15].

Content of each amino acid in the sample:

$$X=\frac{A\times V_{1}\times N}{m\times V_{2}\times 10000}$$

where *X* is the content of each amino acid in the sample in g/100 g; *A* is the mass of amino acids in the sample solution in ng; *V*_1_ is the total volume of the treatment solution in mL; *V*_2_ is the volume of the sample solution used for the determination in mL; *N* is the number of dilution; *m* is the amount of weighing the sample in g.

#### 2.5.3. Nutritional Evaluation of Amino Acids

The following indices were calculated based on the WHO/FAO amino acid standard model [16,17].

Amino acid score (AAS)

(1) $$\mathrm{AAS}/\%=\frac{Some\;kind\;of\;EAA\;content\;in\;protein}{\mathrm{Content}\;\mathrm{of}\;\mathrm{corresponding}\;\mathrm{EAA}\;\mathrm{in}\;\mathrm{FAO}/\mathrm{WTO}}\times 100$$

Essential amino acid index (EAAI)

(2) $$\mathrm{EAAI}/\%=\sqrt{\frac{\mathrm{Th}r_{a}\times \mathrm{Cy}s_{a}\times \mathrm{Me}t_{a}\ldots \ldots \mathrm{Ly}s_{a}}{\mathrm{Th}r_{b}\times \mathrm{Cy}s_{b}\times \mathrm{Me}t_{b}\ldots \ldots \mathrm{Ly}s_{b}}}\times 100$$

Chemical score (CS)

(3) $$\mathrm{CS}/\%=\frac{Relative\;amount\;of\;a\;certain\;EAA\;in\;a\;protein}{\mathrm{Relative}\;\mathrm{content}\;\mathrm{of}\;\mathrm{corresponding}\;\mathrm{EAA}\;\mathrm{in}\;\mathrm{FAO}/\mathrm{WTO}}\times 100$$

Biological value (BV)

(4) BV=1.09×EAAI−11.7

(5) $$\mathrm{RCAA}=\frac{\mathrm{EAA}(specific\;value)}{\mathrm{EAA}(\mathrm{mean}\;\mathrm{value}\;\mathrm{of}\;a\;\mathrm{ratio})}$$

Score of ratio coefficient of amino acid (SRCAA)

(6) SRCAA/%=100−CV×100

Protein efficiency ratio (C-PER)

(7) $$C-\mathrm{PER}=0.08084\times X_{7}-0.1094$$

Protein digestibility corrected amino acid score (PDCAAS)

(8) $$\mathrm{PDCAAS}/\%=\frac{Amino\;acid\;content\;of\;proteins}{\mathrm{Homozygous}\;\mathrm{amino}\;\mathrm{acid}\;\mathrm{content}\;\mathrm{in}\;\mathrm{the}\;\mathrm{FAO}/\mathrm{WTO}\;\mathrm{standard}\;\mathrm{model}}\times \mathrm{In}\;\mathrm{Vitro}\;Digestibility\%$$

Note: In Equations (1) and (2), if the AAS and CS ratios of all essential amino acids are ≥1, the protein is a complete protein and its amino acid score can be regarded as 1; otherwise, the amino acid corresponding to the lowest ratio is a limiting amino acid, and the ratio is the amino acid score; “a” in Equation (3) is the amino acid in the protein sample to be tested, and “b” is the amino acid in the reference protein sample; Equation (4) is the raw material price calculated empirically; CV in Equation (6) is the coefficient of variation of RCAA, and CV = the amino acid score. Amino acid in Equation (3), “a” is the amino acid in the protein sample to be tested, and “b” is the amino acid in the reference protein sample; Equation (4) is the empirically deduced biomass price; CV is the coefficient of variation of the RCAA in Equation (6), and CV = standard deviation/mean; $X_{7}$ is the amino acid of the amino acids Thr, Val, Met, and Isoleucine in Equation (7); and $X_{7}$ is the amino acid of the protein sample to be tested. (Ile), leucine (Leu), phenylalanine (Phe), and lysine (Lys) content sum (g/100 g protein).

### 2.6. Statistical Analysis

All experiments were analyzed with three replications. Results are presented as mean ± standard deviation. Means were compared using one-way ANOVA and Duncan’s test. *p*-values less than 0.05 were considered statistically significant. Statistical analysis was performed using SPSS 19.0 software (SPSS Inc., Chicago, IL, USA). Origin 2021 (OriginLab Institute, Northampton, MA, USA) software was used for graphing.

## 3. Results and Discussion

### 3.1. Protein Digestibility

Protein digestibility is a key nutritional indicator, reflecting the efficiency of protein absorption and its impact on amino acid availability [18]. While animal or human studies are more accurate [19], in vitro simulation provides a rapid, cost-effective, and ethical alternative for its assessment [20]. Hence, this study employed an in vitro simulated gastrointestinal digestion model to determine the protein digestibility.

**Figure 1:**
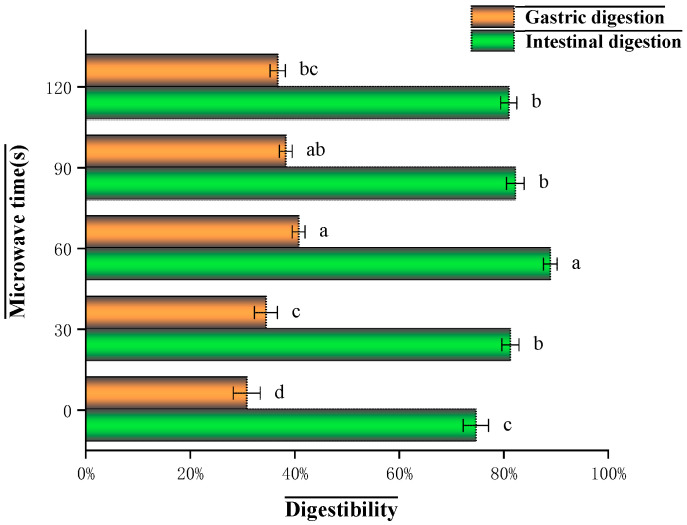
Effect of different microwave treatment times on the digestibility of glycosylated soybean 7S protein. (Bars with different letters are significantly different (*p* < 0.05)).

As shown in Figure 1, the digestibility of glycosylated soybean 7S proteins showed significant differences under different microwave treatment times. In the gastric digestion stage, protein digestibility increased significantly after microwaving. It showed a trend of increasing and then decreasing with the increase in microwave time, reaching the maximum value (40.83%) at 60 s. During the intestinal digestion phase, the trend of protein digestibility is consistent with that in the gastric digestion phase. Both initially increase and then decrease as the microwave treatment time extends, reaching a maximum value (82.37%) at 60 s. Although the digestibility in the 90-s and 120-s groups shows a decline, the intestinal digestibility of all microwave-treated groups (ranging from 81.12% to 82.37%) is significantly higher than that of the untreated control group (81.12% vs. 80.15%). This indicates that microwave pretreatment also significantly improves the digestibility of soybean 7S protein in the intestinal environment. This reduction in digestibility after prolonged microwave exposure could be attributed to excessive structural changes: Extended microwave irradiation promotes the formation of large, insoluble protein aggregates via intensified hydrophobic interactions and disulfide bonds. These dense structures physically shield the interior peptide bonds from access by digestive enzymes like pepsin and trypsin. Given that the sample is glycosylated, prolonged microwave heating provides sustained thermal energy that progressively accelerates the Maillard reaction. The formation of advanced glycation end products (AGEs) is time-dependent; longer microwave exposure offers more time for the initial Schiff base and Amadori products to undergo irreversible rearrangements into these complex, stable cross-links. These AGEs create stable covalent cross-links between protein molecules, which are highly resistant to enzymatic hydrolysis.

Microwave treatment disrupts electrostatic interactions within proteins, exposing otherwise hidden enzyme binding sites. This makes proteins more susceptible to hydrolysis by digestive enzymes such as pepsin and trypsin, thereby improving protein digestibility. Microwave treatment may promote the hydrolysis of proteins, producing more small peptides and amino acids, smaller molecules that are more easily digested and absorbed, improving overall digestibility. Another possible reason is that microwave irradiation may disrupt the structure of heat-labile anti-nutritional factors, such as trypsin inhibitors and lectins, through thermal and non-thermal effects. The denaturation and inactivation of these antinutritional factors (ANFs, e.g., trypsin inhibitors and lectins) eliminate their ability to inhibit proteolytic enzymes or bind to the intestinal mucosa, thereby reducing their interference with the digestion process, improving protein digestibility. Some studies have shown that the in vitro digestibility of proteins is also related to the α-helix and β-fold content of proteins [21]. when the α- helix content is higher than β-fold content, the digestibility of proteins is better [22]. This is consistent with the results of changes in protein secondary structure in the study of Zhang et al. [23]. This study showed that microwaves can significantly improve the digestibility of glycosylated soybean 7S protein.

### 3.2. Hydrolysis

Degree of hydrolysis (DH) represents the percentage of cleaved peptide bonds in a protein, serving as a key indicator of its nutritional quality and bioavailability. A higher DH correlates with enhanced functional properties—such as solubility and emulsification—and improves intestinal absorption of amino acid. The DH is quantified by measuring free amino groups released upon peptide bond cleavage, providing a direct metric of proteolytic extent.

**Figure 2:**
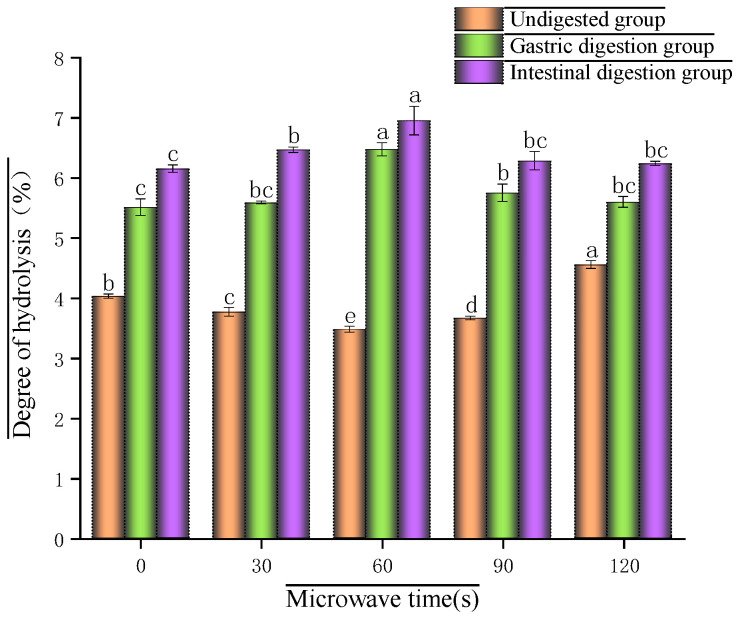
Effect of different microwave treatment times on the hydrolysis degree of glycosylated soybean 7S protein. (Bars with different letters are significantly different (*p* < 0.05)).

When undigested, the hydrolysis degree of glycosylated soybean 7S protein tended to decrease and then increase with the increase in microwave time and reached the maximum value (4.55%) at 120 s (Figure 2). This is because at the initial stage of microwave treatment, the polar groups of protein molecules are affected by microwave radiation, resulting in a change in the polarity of the amino acids. This change may cause the protein molecules to aggregate due to thermal effects, and the molecular structure becomes more compact, which reduces the accessibility of the molecules to the peptide bonds, leading to a decrease in hydrolysis [24]. However, with further extension of microwave time, the excessive temperature denatures the proteins excessively. The internal structure of the protein molecules may be gradually disrupted. The peptide bond breakage increases, exposing more hydrolysis sites, which leads to an increase in the degree of hydrolysis, confirming the previous study’s results. The overall hydrolysis degree of protein increased after gastric and intestinal digestion [25], which were 5.51%, 5.59%, 6.48%, 5.75%, 5.60% (gastric digestion) and 6.15%, 6.47%, 6.95%, 6.29%, 6.24% (intestinal digestion), respectively, suggesting that gastrointestinal digestion facilitates protein hydrolysis, which is in agreement with the study of Cao et al. [26]. However, the microwave time yielding the highest DH shifted from 120 s to 60 s. This apparent contradiction can be explained by the dual effects of microwave treatment: At extended times, excessive thermal energy causes severe protein aggregation, cross-linking (via disulfide bonds and hydrophobic interactions), and advanced Maillard reaction (given the glycosylated nature of our sample). These processes form dense, insoluble structures that physically shield the interior peptide bonds from the access of digestive enzymes (pepsin and trypsin). Consequently, although the microwave itself caused some hydrolysis (high DH in the undigested sample), the resulting aggregated structure became highly resistant to enzymatic attack, leading to a lower final DH after digestion compared to the moderately treated (60 s) sample.

The hydrolysis of glycosylated soybean 7S proteins after microwaving was higher than without, with the highest hydrolysis after 60 s of microwaving. This may be because during gastrointestinal digestion, pepsin and trypsin caused significant changes in the structure of microwaved 7S proteins, which changed from the original ordered structure to disordered, exposing more enzyme binding sites, thus accelerating hydrolysis. In contrast, the non-microwaved 7S protein had been partially unfolded in the initial state, and although the initial hydrolysis degree was higher, its structure changed less during gastric digestion, and the hydrolysis rate was lower than that of the microwaved protein. In the process of structural changes leading to changes in hydrolysis degree, the protein structure at 60 s of microwaving was the most organized and changed the most, so its hydrolysis degree was higher than that of the rest of the protein group at microwave time after digestion.

### 3.3. Amino Acid Composition

Amino acid composition is fundamental to protein nutritional quality, with most assessment methods evaluating efficacy in meeting human amino acid requirements [17]. As shown in Table 2 and Table 3, the hydrolyzed amino acid profile (representing the complete amino acid set after protein breakdown) remained largely consistent across treatments. In contrast, the free amino acid fraction (comprising readily absorbable monomers) showed a notable change: tyrosine, initially undetectable, appeared at a consistent level of approximately 0.11 mg/g following microwave treatment. However, the consistent appearance and level of free tyrosine across all treated samples strongly indicate a clear treatment effect. This suggests that microwave exposure induced structural alterations, releasing previously bound tyrosine. The uniform concentration across treatment times indicates that the release reached a dynamic equilibrium, with prolonged exposure having no further effect.

### 3.4. Nutritional Evaluation of Amino Acids

Establishing an accurate amino acid scoring model is crucial in accurately assessing protein quality. The choice of amino acid scoring model directly determines the accuracy and reliability of protein quality assessment. Currently, several methods are available for evaluating protein quality from food sources, including amino acid scoring (AAS), chemical scoring (CS), essential amino acid index (EAAI), raw material price (BV), amino acid ratio coefficient score (SRCAA), essential amino acid relative ratio (EAARR), protein efficiency ratio (C-PER), and protein digestibility corrected amino acid score (PDCAAS). Nutritional evaluation of glycosylated soybean 7S proteins at different microwave treatment times is shown in Table 4.

The AAS is based on the first limiting essential amino acid (EAA) content in the protein, reflecting its ratio to the EAA content in the FAO/WHO standard protein model. The CS is the score that takes the minimum of the ratio of the relative EAA content of all the proteins to be tested to the relative EAA content in the FAO/WHO standard protein model, both of which focus on assessing the nutritional deficiencies, emphasizing the overall EAA balance of the protein. The AAS was 9.76, 12.91, 23.19, 24.08, and 11.77, and the CS was 14.67, 18.40, 30.41, 30.72, and 17.47, respectively, for different microwave treatment times. It can be found that the AAS and CS of the protein increased and then decreased with the increase in microwave time, and increased rapidly at 60 s and 90 s, which proved that the overall amino acid utilization of glycosylated soybean 7S protein increased at these two time points. EAAI calculates the ratio of all EAA to the FAO/WHO standard protein pattern through the geometric mean method, reflecting the similarity degree of the essential amino acid composition of the tested protein to the FAO/WHO standard protein pattern. A higher index value indicates a more balanced essential amino acid profile, which corresponds to greater nutritional value. Glycosylation, when not microwaved, The EAAI of soybean 7S protein was the lowest at 74.69. The EAAI increased after microwaving and reached the maximum values at 60 and 90 s (88.86 and 88.98), which proved that the amino acid composition of glycosylated soybean 7S protein was more in line with the pattern of amino acid requirement of the human body under these two microwave time conditions. Biological value (BV) refers to the degree of absorption and utilization of ingested proteins by the human body and is expressed as a percentage. Studies have shown that nutrient-rich, high-quality protein foods typically have a BV between 70% and 100% BV, which can be used to assess how efficiently the body utilizes dietary protein. Generally, foods containing higher concentrations of EAA tend to have higher BV [9]. The BV of glycosylated soybean 7S protein increased from 69.71% to 74.88%, 84.29%, 85.94% and 71.71% after microwaving, and exceeded 80% at microwave times of 60 and 90 s, respectively, which proves that its amino acid composition is more compatible with the human body’s needs, and thus is more conducive to the synthesis and metabolism of proteins. SRCAA is a method for statistically evaluating the balance of the EAA ratio in proteins. SRCAA is a statistically significant indicator of the balance of EAA ratios in proteins, based on the coefficient of variation (CV) of the RC value, which reflects the closeness of the amino acid pattern to a standardized pattern such as that of the FAO/WHO. The closer the SRCAA is to 100, the closer the ratio of essential amino acids is to the ideal pattern; if the SRCAA is lower, an imbalance of amino acid ratios (e.g., an over- or under-representation of certain EAAs) exists. The SRCAA of glycosylated soybean 7S protein was the lowest at 82.15 before microwaving but increased after microwaving and reached the maximum at 60 s and 90 s (84.63 and 84.69), which proved that the amino acid composition of glycosylated soybean 7S protein was more in line with the pattern of amino acid requirement in the human body under these two microwave time conditions. C-PER helps to assess the quality of a protein by modeling the efficiency of protein utilization in the body, which reflects the extent to which an organism can efficiently utilize a protein after it has been ingested. Studies have shown that when the C-PER value is below 1.5, it is considered a low-quality protein. This means the organism uses the ingested protein less efficiently [27]. As shown in Table 4, the C-PER values of glycosylated soybean 7S protein before and after microwaving were greater than 1.5, indicating that it maintains good nutritional quality and bioavailability efficiency, and it has high application value, which can effectively support the protein synthesis needs of animals (or the human body).With the increase in microwave time, the C-PER value of the protein increased from 2.26 to 2.68, then decreased to 2.29 at 120 s. This suggests that the microwave treatment time affected the quality and bioavailability of glycosylated soybean 7S protein, and that the moderate treatment could enhance the protein’s digestibility and amino acid availability. Still, the over-treatment might trigger the Melad reaction and thermal degradation, leading to nutritional quality deterioration. Relevant studies have pointed out [17] that in vitro experiments can effectively replace the complex and time-consuming in vivo experiments on large samples of animals, to realize the objective evaluation of the nutritional properties of proteins, which can be measured by simulating the digestive process of the human body, which is not only simple, but also low-cost, and has a high value of application. With PDCAAS, researchers can determine whether a protein can provide a complete amino acid profile for the human btody or whether the protein needs to be combined with other protein sources to meet the human body’s nutritional requirements [28]. In this study, the first limiting amino acid of glycosylated soybean 7S protein was cysteine + methionine, and its PDCAAS at different microwave times were 7.30%, 10.51%, 20.65%, 19.84%, and 9.55%, respectively. The amino acid composition of the proteins was closest to the reference protein pattern, and the PDCAAS value was the highest when the microwave time was 60 s and 90 s. This suggests that these two time points may be the optimal time ranges for microwave treatment, in which the proteins’ amino acid composition and digestibility were favorably altered. The quality of the proteins was improved. Beyond this time range, protein quality will be negatively affected. Excessive microwaving may lead to protein denaturation, amino acid oxidation, or the formation of undigestible compounds (e.g., through advanced Maillard reaction) that act as antinutritional factors, which may reduce the nutritional value of proteins and thus affect the PDCAAS values. Although microwave treatment significantly improved the PDCAAS and other indicators, the PDCAAS value of glycosylated soybean 7S protein (7.30–20.65%) in this study was still significantly lower than that of high-quality animal proteins such as whey protein (PDCAAS ≈ 100%). At the same time, the data shows that the biological value (BV) and predicted protein efficiency ratio (C-PER) are relatively high, but in contrast to the PDCAAS value. This seemingly contradictory phenomenon stems from the different evaluation principles of these indicators. PDCAAS is determined by the score of the first limiting amino acid (the sulfur-containing amino acid in this study), like the ‘wooden barrel effect’, limiting its maximum score. While BV and C-PER are holistic indicators that reflect the overall balance and utilization efficiency of all essential amino acids in the protein. The higher BV and C-PER values in this study indicate that, apart from the sulfur-containing amino acid, the amino acid pattern of glycosylated soybean 7S protein is relatively balanced and easy to be utilized by the body. Therefore, the core value of microwave treatment lies in improving the protein structure, maximizing the alleviation of its inherent limiting amino acid bottleneck, and significantly enhancing its comprehensive nutritional value. This provides an effective strategy for improving the quality and efficiency of plant proteins.

### 3.5. Analysis of Flavor-Presenting Amino Acids

Flavor-presenting amino acids are essential components of food flavor, and they directly or indirectly affect the taste properties of food by binding to taste receptors. Understanding the composition and changing laws of protein flavor-presenting amino acids can help optimize the food processing technology. Free amino acids exist in the form of monomers, can be directly combined with taste receptors (such as taste receptors T1R1/T1R3) to produce freshness, sweetness, or bitterness and other sensory signals, a true reflection of the current flavor of the food, so the free amino acid content can be used to analyze the composition of amino acids in the protein flavor. In the microwave treatment of glycosylated soybean 7S protein, the optimal microwave time can be determined by examining the changes in the content of flavor-presenting amino acids under different microwave times to achieve the desired flavor effect. According to the taste characteristics, the taste amino acids were divided into the following categories: fresh amino acids (aspartic acid Asp and glutamic acid Glu), sweet amino acids (serine Ser, threonine Thr, glycine Gly, alanine Ala and proline Prol), and bitter amino acids (valine Val, methionine Met, leucine Leu, isoleucine Ile, and phenylalanine Phe), the relative contents of the taste amino acids in the glycosylated soybean 7S proteins at different microwave treatment times are shown in Table 5.

As shown in Table 5, the proportion of flavor-presenting amino acid content relative to the total free amino acid content of glycosylated soybean 7S protein was the highest (83.05%) without microwave treatment, indicating that the flavor-presenting amino acids (e.g., aspartic acid, glutamic acid, etc.) of glycosylated soybean 7S protein were more adequately released in the natural state. However, with the extension of microwave time, the proportion of flavor-presenting amino acids decreased significantly (57.18%, 53.77%, 54.28%), which may be attributed to the thermal effect of microwave destroying the protein structure, increasing the release of non-flavor-presenting amino acids (e.g., branched-chain amino acids) or the decomposition of flavor-presenting amino acids. When the microwave time reached 120 s, the proportion of flavor-presenting amino acids increased (62.75%), which may be related to the further hydrolysis of proteins to produce new flavor-presenting substances after microwave.

Among the flavor-presenting amino acids, aspartic acid (Asp) was the most abundant fresh-flavored amino acid, which decreased from 33.41% in the non-microwave-treated group to 16.39% (at 90 s) and then rebounded to 20.92% (at 120 s). Microwaving may initially disrupt the structure of aspartic acid or promote the release of other amino acids, leading to a decrease in the relative proportion. In contrast, more aspartic acid may be released at a later stage due to the deep hydrolysis of the protein. The percentage of glutamic acid (Glu) fluctuated drastically (3% to 13.73%), which may be related to microwaves promoting selective hydrolysis of proteins or the conversion of Glu with other substances (e.g., glutamine). Among the sweet amino acids, the percentages of serine (Ser) and glycine (Gly) showed a fluctuating downward trend (with a brief recovery at 90 s), which may be due to their decomposition or conversion to other substances by microwave. Among the bitter amino acids, the percentage of isoleucine (IIe) increased in the middle stage (9.18%), which might be related to the microwave-promoted protein hydrolysis in the hydrophobic region, releasing more branched-chain amino acids; the decrease in the late stage might be due to further decomposition. The continuous decline in valine (Val) percentage indicates that it is less thermally stable, and microwaves may lead to their decomposition.

**Figure 3:**
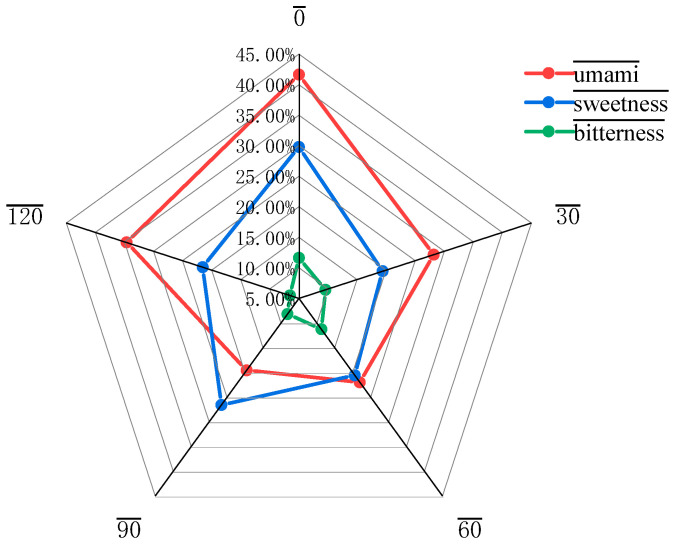
Percentage of flavor-presenting amino acids of glycosylated soybean 7S protein.

Under different microwave times, Figure 3 shows the results of varying microwave treatment times on the percentage of flavor-presenting amino acids in glycosylated soybean 7S protein. At 0–90 s of microwave treatment, the percentage of flavorful amino acids showed a continuous decreasing trend; however, at 120 s of microwave treatment, the percentage rebounded significantly to 55.21%. This phenomenon suggests that a short period of microwave treatment may lead to a decrease in the proportion of fresh and sweet amino acids and a relative increase in the proportion of bitter amino acids in glycosylated soybean 7S proteins, resulting in a certain degree of deterioration in flavor quality. In the later stages of the microwave treatment, the proportions of the flavor components gradually recovered. However, the overall proportions of fresh and sweet amino acids were still significantly lower than the initial level.

As can be seen from Table 6, the TAV of fresh flavor amino acids (Asp, Glu) increased at 30 s and 120 s compared with 0 s, but were lower than those of the un-microwaved group (0 s) at 60 s and 90 s. The TAV of the sweet amino acids (Gly, Ser, Thr) were lower than those of the un-microwaved group (0 s). The TAV of sweet amino acids (Gly, Ser, Thr) fluctuated less with the increase in microwave time, and some sweet amino acids (Ala, Pro) were n.d., indicating that the microwave treatment time had little effect on the TAV of sweet amino acids. Among the bitter amino acids, the TAV of valine (Val) was higher at 30 s and 120 s (0.0325), and the TAV of isoleucine (IIe) was highest at 60 s (0.031). Overall, the TAV of all amino acids were less than 1, indicating a limited direct impact on flavor perception at the concentrations measured. This preliminary conclusion based on TAV analysis should be confirmed by sensory evaluation in future studies to account for potential synergistic effects, the free amino acid content can be improved by process optimization or auxiliary technology to achieve significant flavor enhancement [29].

## 4. Conclusions

This study provides a clear operational framework for leveraging microwave technology to enhance the value of glycosylated soybean 7S protein. The results demonstrate that microwave treatment time is a decisive factor creating a functional trade-off: a 60-s treatment at 900 W was identified as the optimal window for maximizing nutritional quality. This specific protocol significantly improved in vitro protein digestibility and key nutritional indices, including the essential amino acid index (EAAI) and the protein digestibility-corrected amino acid score (PDCAAS), by inducing structural changes that facilitate enzymatic hydrolysis.

However, this nutritional enhancement was accompanied by a reduction in the relative proportion of flavor-presenting amino acids, indicating a potential compromise in taste profiles. Despite this, the overall amino acid balance and utilization efficiency (as reflected by the biological value) were superior at the 60-s mark.

In conclusion, this research delivers a practical and efficient strategy for the food industry to tailor the functional properties of plant proteins. By adopting the precise microwave parameters outlined here (60 s, 900 W), manufacturers can develop soy protein-based ingredients with significantly improved bioavailability for applications in sports nutrition, health foods, and dietary supplements. Future research can focus on complementary techniques, such as flavor masking or encapsulation, to address the flavor aspect while retaining the nutritional benefits achieved through microwave processing.

## Figures and Tables

**Table 1:** Preparation of simulated gastric fluid (SGF) and simulated intestinal fluid (SIF) stock solutions.

Reagents	Stock Conc.	SGF		SIF	
	moL/L	mL	mmoL/L	mL	mmoL/L
KCl	0.5	6.9	6.9	6.8	6.8
KH_2_PO_4_	0.5	0.9	0.9	0.8	0.8
NaHCO_3_	1	12.5	25	42.5	85
NaCl	2	11.8	47.2	9.6	38.4
MgCl_2_(H_2_O)_6_	0.15 L	0.4	0.1	1.1	0.33
(NH_4_)_2_CO_3_	0.5	0.5	0.5		
pH		3		7	
ultrapure water		400 mL		400 mL	

**Table 2:** Effect of different microwave times on hydrolyzed amino acid content of glycosylated soybean 7S protein (unit: mg/g).

Hydrolyzed Amino Acids					
amino acid type	0 s	30 s	60 s	90 s	120 s
aspartic acid(Asp)	90.8	93.2	100	103	92.7
threonine(Thr)	29.4	32.3	34.6	35.7	29.8
serine(Ser)	40.6	41.9	45	44.8	41.6
glutamic acid(Glu)	148	136	147	148	151
glycine(Gly)	31.2	32.5	34.9	35.7	31.2
alanine(Ala)	32.7	35	37.4	38.2	33.1
cysteine(Cys)	2.4	3.3	4.3	3.4	1.7
valine(Val)	40.2	42.2	45.1	46.3	40.1
methionine(Met)	1	1.2	3.8	5	2.4
isoleucine(IIe)	34.4	35.5	38	39.2	34.6
leucine(Leu)	60.4	64.7	69.8	71.6	61.1
tyrosine(Tyr)	24.4	27.6	29.9	30.2	24.2
phenylalanine(Phe)	40.2	42.7	46	47.4	41
lysine(Lys)	60.2	59	63.8	65.9	61.3
histidine(His)	18.8	19.6	21.2	21.8	18.8
arginine(Arg)	65.7	66.1	71.2	73.1	66.4
proline(Pro)	31.9	35	36.6	37.6	32.3
EAA	292.6	308.5	335.3	344.7	296.2
NEAA	459.7	459.3	493.3	502.2	467.1
TAA	752.3	767.8	828.6	846.9	763.3
EAA/TAA	0.39	0.40	0.40	0.41	0.39
NEAA/TAA	0.61	0.60	0.60	0.59	0.61
EAA/NEAA	0.64	0.67	0.68	0.69	0.63

**Table 3:** Effect of different microwave times on free amino acid content of glycosylated soybean 7S protein (unit: mg/g).

Free Amino Acids					
amino acid type	0 s	30 s	60 s	90 s	120 s
aspartic acid(Asp)	0.069	0.074	0.059	0.057	0.096
threonine(Thr)	0.0085	0.014	0.0057	0.0084	0.018
serine(Ser)	0.029	0.035	0.036	0.056	0.048
glutamic acid(Glu)	0.017	0.032	0.008	0.011	0.063
glycine(Gly)	0.024	0.024	0.021	0.028	0.033
alanine(Ala)	N.d. (<0.0097)	N.d. (<0.0097)	N.d. (<0.0097)	N.d. (<0.0097)	N.d. (<0.0097)
cysteine(Cys)	0.015	0.019	0.015	0.029	0.019
valine(Val)	0.008	0.013	0.0063	0.0074	0.013
methionine(Met)	N.d. (<0.0075)	N.d. (<0.0075)	N.d. (<0.0075)	N.d. (<0.0075)	N.d. (<0.0075)
isoleucine(IIe)	0.016	0.023	0.028	0.021	0.017
leucine(Leu)	N.d. (<0.0036)	N.d. (<0.0036)	N.d. (<0.0036)	N.d. (<0.0036)	N.d. (<0.0036)
tyrosine(Tyr)	N.d. (<0.0095)	0.11	0.11	0.11	0.11
phenylalanine(Phe)	N.d. (<0.0083)	N.d. (<0.0083)	N.d. (<0.0083)	N.d. (<0.0083)	N.d. (<0.0083)
lysine(Lys)	0.02	0.032	0.016	0.02	0.042
histidine(His)	N.d. (<0.0020)	N.d. (<0.0020)	N.d. (<0.0020)	N.d. (<0.0020)	N.d. (<0.0020)
arginine(Arg)	N.d. (<0.0065)	N.d. (<0.0065)	N.d. (<0.0065)	N.d. (<0.0065)	N.d. (<0.0065)
proline(Pro)	N.d. (<0.0087)	N.d. (<0.0087)	N.d. (<0.0087)	N.d. (<0.0087)	N.d. (<0.0087)
EAA	0.0675	0.211	0.181	0.1958	0.219
NEAA	0.139	0.165	0.124	0.152	0.24
TAA	0.2065	0.376	0.305	0.3478	0.459
EAA/TAA	0.33	0.56	0.59	0.56	0.48
NEAA/TAA	0.67	0.44	0.41	0.44	0.52
EAA/NEAA	0.49	1.28	1.46	1.29	0.91

N.d.: not detected.

**Table 4:** Nutritional evaluation of glycosylated soybean 7S protein at different microwave times.

Nutritional Assessment	Unit	0 s	30 s	60 s	90 s	120 s
AAS	%	9.76	12.91	23.19	24.08	11.77
CS	%	14.67	18.40	30.41	30.72	17.47
EAAI	%	74.69	79.43	88.06	89.58	76.52
SRCCA		82.15	83.10	84.63	84.69	82.43
C-PER		2.26	2.39	2.60	2.68	2.29
BV	%	69.71	74.88	84.29	85.94	71.71
PDCAAS	%	7.30	10.51	20.65	19.84	9.55

**Table 5:** Relative contents of flavor-presenting amino acids in glycosylated soybean 7S protein under different microwave treatment times.

Free Amino Acids					
types of amino acids	0 s	30 s	60 s	90 s	120 s
aspartic acid (Asp)	33.41	19.68	19.34	16.39	20.92
threonine (Thr)	4.12	3.72	1.87	2.42	3.92
serine (Ser)	14.04	9.31	11.80	16.10	10.46
glutamic acid (Glu)	8.23	8.51	2.62	3.16	13.73
glycine (Gly)	11.62	6.38	6.89	8.05	7.19
alanine (Ala)	/	/	/	/	/
cysteine(Cys)	7.26	5.05	4.92	8.34	4.14
valine (Val)	3.87	3.46	2.07	2.13	2.83
methionine (Met)	/	/	/	/	/
isoleucine (IIe)	7.75	6.12	9.18	6.04	3.70
leucine (Leu)	/	/	/	/	/
tyrosine (Tyr)	/	29.26	36.07	31.63	23.97
phenylalanine (Phe)	/	/	/	/	/
lysine (Lys)	9.69	8.51	5.25	5.75	9.15
histidine (His)	/	/	/	/	/
arginine (Arg)	/	/	/	/	/
proline (Pro)	/	/	/	/	/
taste TAA/TAA	83.05	57.18	53.77	54.28	62.75

**Table 6:** TAV of glycosylated soybean 7S protein at different microwave times (unit: mg/g).

Types of Amino Acids		Threshold	TAV				
			0 s	30 s	60 s	90 s	120 s
umami	aspartic acid (Asp)	1	0.0690	0.0740	0.0590	0.0570	0.0960
	glutamic acid (Glu)	0.3	0.0567	0.1067	0.0267	0.0367	0.2100
sweet taste	glycine (Gly)	1.3	0.0185	0.0185	0.0162	0.0215	0.0254
	alanine (Ala)	0.6	N.d. (<0.0020)	N.d. (<0.0020)	N.d. (<0.0020)	N.d. (<0.0020)	N.d. (<0.0020)
	proline (Pro)	3	N.d. (<0.0020)	N.d. (<0.0020)	N.d. (<0.0020)	N.d. (<0.0020)	N.d. (<0.0020)
	serine (Ser)	1.5	0.0193	0.0233	0.0240	0.0373	0.0320
	threonine (Thr)	2.6	0.0033	0.0054	0.0022	0.0032	0.0069
bitter taste	Valine (Val)	0.4	0.0200	0.0325	0.0158	0.0185	0.0325
	isoleucine (IIe)	0.9	0.0178	0.0256	0.0311	0.0233	0.0189
	leucine (Leu)	1.9	N.d. (<0.0020)	N.d. (<0.0020)	N.d. (<0.0020)	N.d. (<0.0020)	N.d. (<0.0020)
	histidine (His)	0.2	N.d. (<0.0020)	N.d. (<0.0020)	N.d. (<0.0020)	N.d. (<0.0020)	N.d. (<0.0020)
	arginine (Arg)	0.5	N.d. (<0.0020)	N.d. (<0.0020)	N.d. (<0.0020)	N.d. (<0.0020)	N.d. (<0.0020)
	methionine (Met)	0.3	N.d. (<0.0020)	N.d. (<0.0020)	N.d. (<0.0020)	N.d. (<0.0020)	N.d. (<0.0020)
	phenylalanine (Phe)	0.9	N.d.(<0.0020)	N.d.(<0.0020)	N.d.(<0.0020)	N.d.(<0.0020)	N.d.(<0.0020)

N.d.: not detected.

## Data Availability

The original contributions presented in this study are included in the article. Further inquiries can be directed to the corresponding authors.

## Abbreviations

The following abbreviations are used in this manuscript:

SGF	simulated gastric fluid
SIF	simulated intestinal fluid
AAS	amino acid score
CS	chemistry scoring
EAAI	essential amino acid index
BV	commodity prices
SRCAA	score of the ratio coefficient of the amino acid
C-PER	protein efficiency ratio
PDCAAS	protein digestibility corrected amino acid score
DH	hydrolysis
EAA	essential amino acid
NEAA	non-essential amino acids
TAA	total amino acids
